# ﻿*Cirrhimuraenataiwanensis* sp. nov., a new species of cirri-bearing eel (Anguilliformes, Ophichthidae) from Yilan, northeastern Taiwan

**DOI:** 10.3897/zookeys.1224.141248

**Published:** 2025-01-24

**Authors:** Yen-Ting Lin, Yu-Hsiang Lin, Yu-San Han

**Affiliations:** 1 Institute of Fisheries Science, College of Life Science, National Taiwan University, Taipei, Taiwan National Taiwan University Taipei Taiwan

**Keywords:** Biodiversity, brackish water, COI analysis, Taiwanese fringe-lip eel, taxonomy

## Abstract

A new species of cirri-bearing eel, *Cirrhimuraenataiwanensis***sp. nov.** (Anguilliformes, Ophichthidae), is described based on a specimen collected from the estuary of the Langyang River (Yilan County), northeastern Taiwan. The new species is distinct from all congeners, except *C.odishaensis* and *C.orientalis*, in possessing a single row of mandibular teeth. *Cirrhimuraenataiwanensis***sp. nov.** differs from *C.odishaensis* in having significantly shorter pectoral fins and fewer vertebrae, and it is distinguished from *C.orientalis* by its larger head, notably more total vertebrae, and a dorsal fin that originates well behind the gill opening. In the neighbor-joining tree based on COI sequences, the new species forms a distinct monophyletic group; thus, it is clearly separable from congeners both morphologically and genetically. With this addition, there are now 13 species in the genus *Cirrhimuraena*.

## ﻿Introduction

The family Ophichthidae, commonly known as snake eels, represents the most varied group within the order Anguilliformes, containing two subfamilies (Myrophinae and Ophichthinae), with 62 genera and 361 species recorded (Fricke et al. 2024). In Taiwan, there have been reported of 19 genera and 60 species of snake eels identified (Chiu et al. 2018).

The genus *Cirrhimuraena* Kaup, 1856 belongs to the subfamily Ophichthinae and is known as cirri-bearing eels. This genus is notable for its unique morphological traits, particularly the presence of cirri, which are small, fleshy projections located on the upper jaw (Mohapatra et al. 2021). Cirri-bearing eels typically inhabit sandy or muddy substrates in coastal and estuaries waters (Mohapatra et al. 2021; Mohanty et al. 2023). Currently, 12 valid species are known, including *Cirrhimuraenachinensis* (Kaup, 1856), *C.tapeinoptera* (Bleeker, 1863), *C.cheilopogon* (Bleeker, 1860), *C.calamus* (Günther, 1870), *C.playfairii* (Günther, 1870), *C.oliveri* (Seale, 1910), *C.paucidens* (Herre & Myers, 1931), *C.inhacae* (Smith, 1962), *C.orientalis* (Nguyen, 1993), *C.yuanding* (Tang & Zhang, 2003), *C.indica* (Mohapatra, Mohanty, Ray, Mishra & Seth, 2021), and *C.odishaensis* (Mohanty, Behera, Patro & Mohapatra, 2023). Of these 12 species, only one, *C.chinensis*, has been recorded in Taiwan, and research on this genus remains scarce (Ho et al. 2015).

We conducted a survey of freshwater glass eels (juveniles of *Anguilla* spp.) in the the Langyang River estuary in northeastern Taiwan (24.7162°N, 121.8352°E) twice a month since 2010. Notably, this survey has yielded both a new species of Ophichthidae (*Lamnostomataiwanense* Chiu, Huang & Shao, 2018) and new records of *Anguillaborneensis* Popta, 1924 and *A.interioris* Whitley 1938 (Chiu et al. 2018; Lin and Han 2024). In December 2023, a single *Cirrhimuraena* specimen was collected. A morphological analysis and molecular evidence indicated that this specimen represents an undescribed species. Although only a single specimen was obtained, its distinct morphology and genetic characteristics underscore its importance in advancing our understanding of *Cirrhimuraena* species in Taiwan. Furthermore, the discovery of new species and fish records highlights the need for conservation efforts to protect fish biodiversity in the Langyang River, one of the most critical habitats for Anguilliformes in Taiwan (Han et al. 2016).

## ﻿Materials and methods

### ﻿Sample collection

The specimen was collected from the estuary of the Langyang River in Yilan County, Taiwan (24.7162°N, 121.8352°E) on December 22, 2023. The environmental conditions of the collection site at the time of collection were as follows: substrate sandy, water depth 1 m, salinity 7‰, and water temperature 18 °C. A single, undescribed specimen of *Cirrhimuraena* was captured using a fyke net. Once collection, the specimen was photographed and radiographed, measured, and subsequently preserved in 95% ethanol. The specimen was deceased at the time of collection, and no live animals were included in this study.

### ﻿Measurement and comparisons

The morphometrics were measured with digital calipers with an accuracy of 0.1 mm, and the meristic analysis and counting of head pores followed the protocol used by McCosker et al. (1989). The identified specimen was deposited in the collection of Biodiversity Research Museum of the Academia Sinica of Taiwan (**ASIZP**) under registration code ASIZP0082637. The specimen was compared with records of all congeners documented from Taiwan, *Cirrhimuraenachinensis*, and nearby waters, including *C.yuanding* from Pingtan, China (Tang and Zhang 2003), and *C.playfairii* from Okinawa, Japan (Hibino et al. 2021).

### ﻿Molecular analysis

The dorsal muscle was dissected for the total genomic DNA extraction using the EasyPure Genomic DNA Spin Kit (Bioman Scientific, Taiwan). A polymerase chain reaction (PCR) was carried out to amplify the partial segment of the cytochrome c oxidase subunit I (COI) by using the forward primer FishF1+2 (5′-TCR ACY AAY CAY AAA GAY ATY GGC AC-3′) and the reverse primers FishR1 (5′-TAG ACT TCT GGG TGG CCA AAG AAT CA-3′) and FishR2 (5′-ACT TCA GGG TGA CCG AAG AAT CAG AA-3′) following the protocol adjusted from Chang et al. (2016). The final PCR product was sequenced using the primer FishF1+2 by Genomics Scientific, Taiwan.

The COI sequences were aligned and trimmed using BioEdit v. 7.7.1, resulting in partial sequences of 562 base pairs. Once aligned, the sequences were saved in FASTA format and imported into MEGA v. 11 (Tamura et al. 2021) for phylogenetic analysis. A neighbor-joining (NJ) tree was constructed using the Kimura 2-parameter (K2P) distance model, with 10,000 bootstrap replicates to assess the reliability of the branches. Among all congeners, only two valid species of *Cirrhimuraena*, *C.chinensis* and *C.indica*, were available in the NCBI GenBank. In total, 16 sequences were used to construct the NJ tree. In the ingroup, *C.chinensis* (GenBank numbers KY472820.1, KX215192.1, KX215193.1, KX215194.1, MK264639.1, MK264640.1, MK264641.1, GU674221.1, and GU674224.1), *C.indica* (GenBank number MT019886.1), and *C.taiwanensis* sp. nov. (GenBank number PQ558516.1) were included. In the outgroup, and following the study by Mohapatra et al. (2021) were *Ophichthuslithinus* Jordan & Richardson, 1908 (GenBank number KU94289.1), *O.zophochir* Jordan & Gilbert, 1882 (GenBank number GU440436.1), *O.olivaceus* McCosker & Bogorodsky, 2020 (GenBank number MN480448.1), and *Pisodonophiscancrivorus* Richardson, 1848 (GenBank number KU942788.1, and MK777102.1). The details of all COI sequences used are listed in Table 1.

**Table 1.** The detail of the sequences used in the phylogenetic analysis.

Species	NCBI Accession number	Source	Voucher Number	Sampling Locality
*Cirrhimuraenataiwanensis* sp. nov.	PQ524198.1	This study	ASIZP0082637	Yilan, Taiwan
*Cirrhimuraenachinensis*	KY472820.1	GenBank	PT011	China
*Cirrhimuraenachinensis*	KX215192.1	GenBank	JLJ050	China
*Cirrhimuraenachinensis*	KX215193.1	GenBank	JLJ051	China
*Cirrhimuraenachinensis*	KX215194.1	GenBank	JLJ052	China
*Cirrhimuraenachinensis*	MK264639.1	GenBank	PTD055	China
*Cirrhimuraenachinensis*	MK264640.1	GenBank	PT055	China
*Cirrhimuraenachinensis*	MK264641.1	GenBank	QZ053	China
*Cirrhimuraenachinensis*	GU674221.1	GenBank	BWA6863	Indonesia
*Cirrhimuraenachinensis*	GU674224.1	GenBank	BWA6862	Indonesia
*Cirrhimuraenaindica*	MT019886.1	GenBank	EBRC/ZSI/11811	India
*Ophichthuslithinus*	KU942789.1	GenBank	ASIZP0801626	Taiwan
*Ophichthusolivaceus*	MN480448.1	GenBank	KAU17-80	Saudi Arabia
*Ophichthuszophochir*	GU440436.1	GenBank	MFC132	California, USA
*Pisodonophiscancrivorus*	MK777102.1	GenBank	DOS05154	Vietnam
*Pisodonophiscancrivorus*	KU942788.1	GenBank	ASIZP0800053	Taiwan

## ﻿Results

**Family Ophichthidae**

### ﻿ Cirrhimuraena taiwanensis sp. nov.

Taxon classificationAnimaliaAnguilliformesOphichthidae

9A42C280-2671-5596-A5BB-6E2E6434C524

https://zoobank.org/3214769E-E179-46DD-B800-CE12D2A318B0

**Figure 1.**
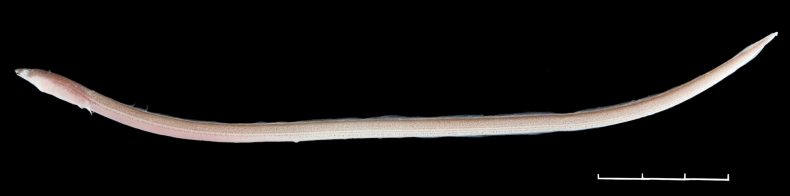
*Cirrhimuraenataiwanensis* sp. nov., ASIZP0082637, 178.1 mm total length. Scale bar: 30 mm.

**Figure 2.**
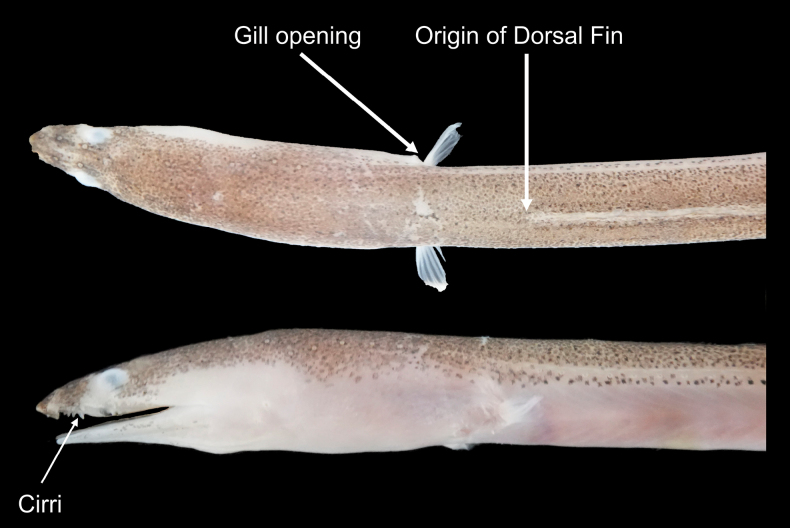
Head profile of *Cirrhimuraenataiwanensis* sp. nov. **A** origin of dorsal fin well behind gill opening **B** arrow indicates cirri on upper jaw.

**Figure 3.**
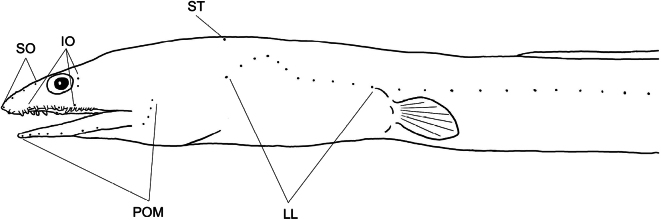
Head and lateral line pores in *Cirrhimuraenataiwanensis* sp. nov. Abbreviaitons: IO: infraorbital pores; LL: lateral-line pores; POM: preoperculomandibular pores; SO: supraorbital pores; ST: supra-temporal pores.

**Figure 4.**
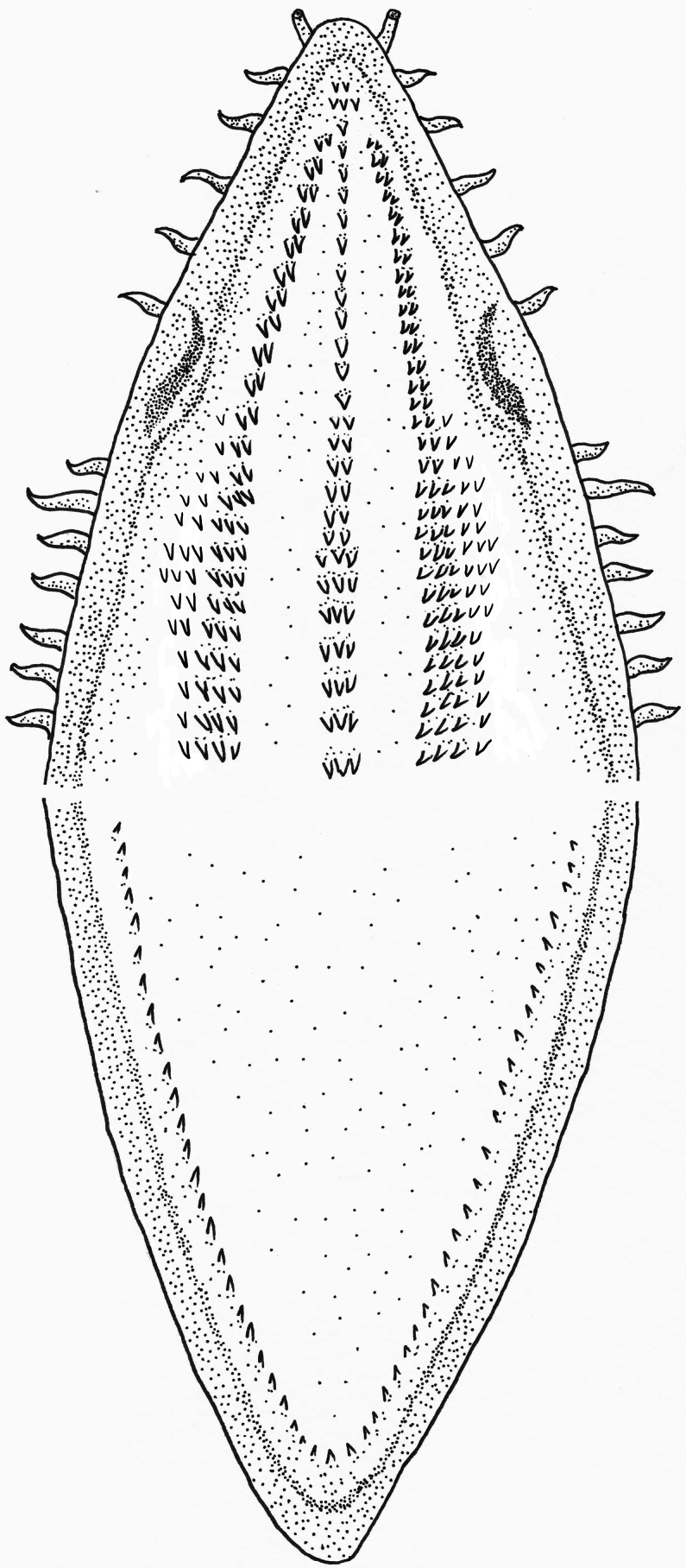
Tooth dentition pattern in upper and lower jaws of *Cirrhimuraenataiwanensis* sp. nov. (holotype, ASIZP0082637, 178.1 mm total length).

Figs 1 , 2 , 3 , 4 , Table 2

#### Material examined.

***Holotype***: Taiwan • ASIZP0082637, 178.1 mm total length (TL); Yilan; 24.7162°N, 121.8352°E; 22 Dec. 2023; caught by fyke net, ca 1 m, Yu-San Han & Yen-Ting Lin leg.

#### Diagnosis.

A new *Cirrhimuraena* species with the combination of following characteristics: pectoral fin very small, only 15.2% of head length (HL) (in congeners > 21% HL); HK 9.7% of TL dorsal fin originates 1½ pectoral-fin length behind gill opening; tooth pattern unique, with only a single row of mandibular teeth; cirri on upper jaw 11; vertebrae 150, vertebral formula 13-53-150.

#### Description.

The morphometric and meristic measurements of the holotype are shown in Table 2. Body very elongate, cylindrical; body height is almost consistent from gill opening to anus, with depth at gill opening of 2.2% of TL and depth at anus of 2.3% of TL. Head moderate, with head length (HL) 9.7% of TL. Tail longer than trunk, 63.6% of TL. Anal fin low, situated right after anus, with pre-anal length 36.8% of TL. Dorsal fin also low, originating far behind gill opening and pectoral fin; pre-dorsal length 13.2% of TL. Pectoral fin very small, 15.2% of HL, 1.5% of TL; pectoral-fin base positioned at same vertical as gill opening; gill opening positioned on latero-ventral side, length 16.4% of HL.

**Table 2.** Morphometric and meristic data of *Cirrhimuraenataiwanensis* sp. nov.

	*Cirrhimuraenataiwanensis* sp. nov. Holotype, *ASIZP0082637*
**Total length (SL, mm)**	178.1
**Head length (HL, mm)**	17.4
**Pre-anal length (PAL, mm)**	65.6
**Pre-dorsal length (PDL, mm)**	23.6
% ***in HL***	
Snout length	19.1
Eye diameter	8.9
Interorbital length	6.1
Upper jaw length	35.7
Lower jaw length	26.9
Gill opening length	16.4
Pectoral-fin length	15.2
% ***in TL***	
Head length	9.7
Pre-anal length	36.8
Pre-dorsal length	13.2
Trunk length	27.1
Tail length	63.6
Depth at gill opening	2.2
Depth at anus	2.3
**Pores**	
Supraorbital	1 + 3
Infraorbital	3 + 2
Preoperculomandibular	7 + 4
Pores before pectoral fin	11
Pores before dorsal fin	16
Pores before anus	48
**Vertebrae**	
Pre-dorsal	13
Pre-anal	53
Total	150

Eye relatively large, positioned nearer to snout tip than rictus; eye diameter 8.9% of HL; interorbital space slightly wider; interorbital length 6.1% of HL. Anterior nostril tubular, positioned at snout tip, while posterior nostril lies slightly behind orbit. Snout long, pointed, 19.1% of HL. Upper jaw longer than lower jaw, 35.7% and 26.9% of HL, respectively.

Five small cirri on edge of upper jaw between anterior and posterior nostrils; 6 cirri behind posterior nostril. No cirri on lower jaw and tip of jaw in front of nostrils. Dentition pattern illustrated in Fig. 3. Teeth numerous, closely arranged in a band, and primarily small and pointed, with slightly larger teeth at ends. Vomerine teeth in 1–3 rows, extending to posterior of maxilla; 5 teeth form a small patch at prevomer. Maxillary teeth in 2–6 rows of small, conical teeth; mandibular teeth band in only a single row on both sides. Pre-dorsal vertebrae 13, pre-anal vertebrae 53, and total vertebral 150.

Head pores tiny and indistinct, with supraorbital pores 1 + 3, infraorbital pores 3 + 2, preoperculomandibular pores 7 + 4, and supra-temporal pores 1 (Fig. 4). Lateral line pores before pectoral fin/gill opening 12, before dorsal-fin origin 16, and before anus 48.

Dorsal surface of body grayish, with numerous tiny black spots; some melanophores concentrated at tip of snout. Ventral side whitish. Dorsal and anal fins translucent; pectoral fin whitish.

#### Distribution.

Currently only known from the type locality, with sandy substrate.

#### Etymology.

The specific epithet *taiwanensis* refers to the location of the type locality, which recently only known in Taiwan; it is used as an adjective.

#### Remarks.

Compared to all 12 congeners, *C.taiwanensis* sp. nov. can be easily distinguished from 10 species, except *C.odishaensis* and *C.orientalis*, in having only a single row of mandibular teeth (Fig. 4). However, *C.taiwanensis* sp. nov. can be separated from these two species morphologically, with comparative details shown in Table 3. The new species differs from *C.odishaensis* in having a shorter pectoral fin, only 15.2% of HL (compared to 21.3–25.0% HL in *C.odishaensis*); fewer vertebrae, with 13 pre-dorsal, 53 pre-anal, and 150 total vertebrae (vs 10 pre-dorsal, 46–47 pre-anal, and 160–162 total vertebrae in *C.odishaensis*); and fewer rows of maxillary teeth (2–6 rows in *C.taiwanensis* sp. nov. vs 3–7 rows in *C.odishaensis*). Compared to *C.orientalis*, *C.taiwanensis* sp. nov. has a larger head at 9.7% of TL (vs 5.5–6.2% of TL in *C.orientalis*), significantly more vertebrae (150 vs 131–136 in *C.orientalis*), and more rows of maxillary teeth (2–6 rows in *C.taiwanensis* sp. nov. vs 2–3 rows in *C.orientalis*).

**Table 3.** Morphometric comparisons of *Cirrhimuraenataiwanensis* sp. nov. with congeners with only a single row of mandibular teeth.

	*Cirrhimuraenataiwanensis* sp. nov. (This study)	*C.odishaensis* (Mohanty et al., 2023)	*C.orientalis* (Nguyen., 1993)
HL % in TL	9.7	9.1–10.6	5.5–6.2
Pectoral fin % in HL	15.2	21.3–25.0	—
Mandibular teeth	1 row	1 row	1 row
Maxillary teeth	2–6 rows	3–7 rows	2–3 rows
Total vertebrate	150	160–162	131–136

— No data available.

#### Molecular results.

**Figure 5.**
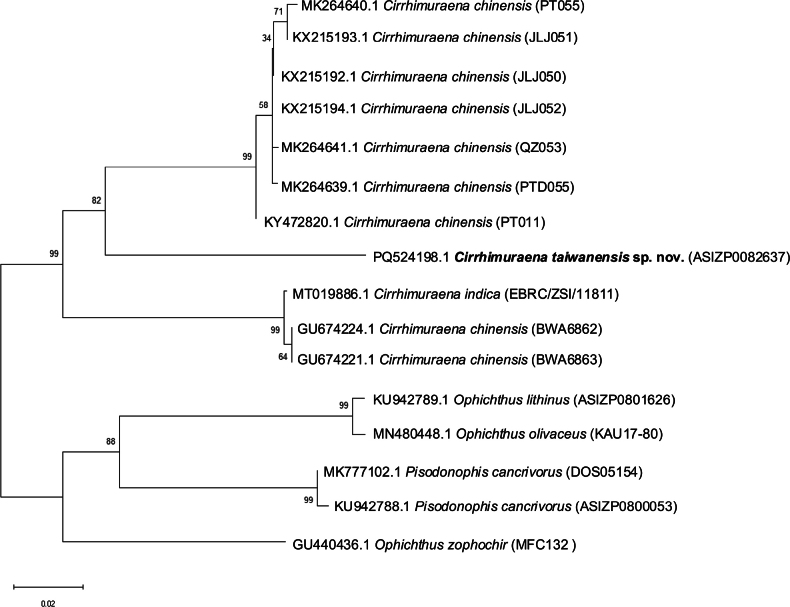
The neighbor-joining tree based on COI sequences of *Cirrhimuraenataiwanensis* sp. nov. and all the valid congeners in NCBI.

Sixteen COI sequences from three taxa were analyzed, revealing nine unique haplotypes across 562 aligned base pairs, which included 196 variable sites and 151 parsimony-informative sites. The NJ tree analysis identified *C.taiwanensis* sp. nov. in a well-supported clade (bootstrap values 99%) with all other *Cirrhimuraena* species included in NCBI (Fig. 5). The average pairwise K2P genetic distance between *C.taiwanensis* sp. nov. and its congeners is 0.124, aligning with the average genetic distance typically found among congeneric fish species, as reported by Ward et al. (2005). Within the *Cirrhimuraena* group, *C.taiwanensis* sp. nov. and most *C.chinensis* specimens are clearly separated (bootstrap value 99%) from *C.indica* and two *C.chinensis* specimens collected in Indonesia (GU674221.1 and GU674224.1), which are suspected misidentifications of *C.indica* (Mohapatra et al. 2021). *Cirrhimuraenataiwanensis* sp. nov. also demonstrates a distinct separation from *C.chinensis*, with high bootstrap support of 82% and forming a unique monophyletic group. The distinct morphological characteristics and NJ tree results further support the separation of *C.taiwanensis* sp. nov. as a distinct species.

## ﻿Discussion

**Figure 6.**
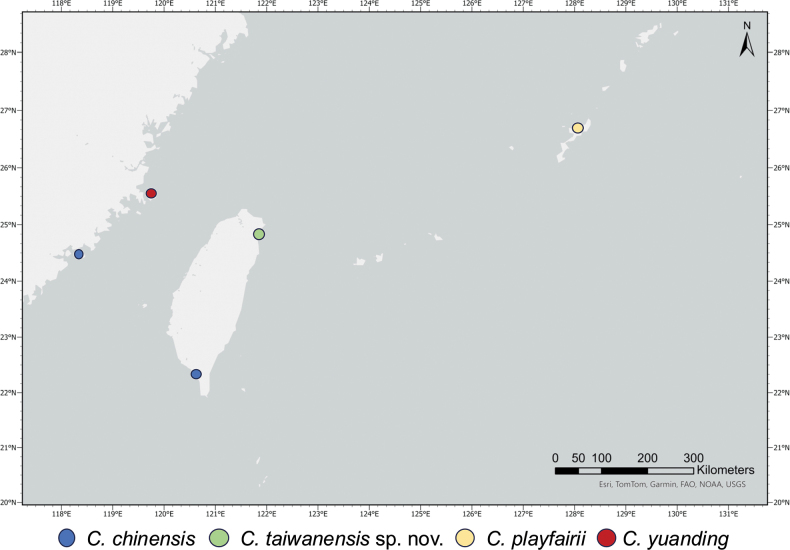
Distribution map of *Cirrhimuraena* species found in Taiwanese waters and nearby regions.

Currently, there are 12 valid species in the genus *Cirrhimuraena*, and the distribution in the northwestern Pacific Ocean is primarily centered around the South China and Java Seas (Mohapatra et al. 2021; Mohanty et al. 2023). Only one species, *C.chinensis*, has been recorded from Taiwanese waters, from along the coast of Pingtung in southwestern Taiwan and Kinmen Island (Shao et al. 2008; Ho et al. 2015). Additional to *C.chinensis* recorded in Taiwanese waters, in the subtropical North Pacific there are two additional species: *C.playfairii*, recorded from Makiya, Okinawa Island, Japan (Hibino et al. 2021: fig. 6b, c), and *C.yuanding*, recorded from Pingtan County, Fujian Province, China (Tang and Zhang 2003) (Fig. 6).

In Table 4, we compare the new species, which exhibits distinct morphological differences from congeners found in Taiwanese waters (*C.chinensis*) and nearby regions (*C.yuanding* and *C.playfairii*). The two species recorded from China (*C.yuanding*) and Japan (*C.playfairii*) can be clearly distinguished from *C.taiwanensis* sp. nov. by the position of the dorsal fin, which originates in front of the gill opening in both *C.playfairii* and *C.yuanding*, with a pre-dorsal length (PDL) shorter than the HL (Table 4). Compared to the *C.chinensis*, *C.taiwanensis* sp. nov. has a significantly shorter pectoral fin at 15.2% of HL (vs 45.2–51.6% HL in *C.chinensis*); a smaller gill opening length at 16.4% of HL (vs 25.4–30.1% HL in *C.chinensis*); dorsal fin that originates well behind the gill opening, with a PDL of 13.2% TL (vs 9.6–11.2% TL in *C.chinensis*); and a slightly smaller head, at 9.7% TL (vs 10.9–11.8% TL in *C.chinensis*) (Table 4). Furthermore, molecular data confirm the distinction between *C.chinensis* and *C.taiwanensis* sp. nov., with a high bootstrap value (82%) supporting their separation (Fig. 5).

**Table 4.** Comparisons of *Cirrhimuraenataiwanensis* sp. nov. and *C.chinensis* in Taiwan, and two other congeners recorded from nearby waters.

	*Cirrhimuraenataiwanensis* sp. nov. Holotype	*C.chinensis n = 10*	*C.yuanding n* = 1 (Tang and Zhang 2003)	*C.playfairii n* = 1 (Hibino et al. 2021)
**Collection site**	Taiwan	Taiwan	China	Japan (Okinawa)
**Total length (SL, mm)**	178.1	227–293	520.5	229
**Head length (HL, mm)**	17.4	23.5–28.9	30.0	—
**Pre-anal length (PAL, mm)**	65.6	89.3–94.2	161.3	—
**Pre-dorsal length (PDL, mm)**	23.6	25.5–28.2	20.5	—
% ***in HL***				
Snout length	19.1	18.9–22.7	16.0	15.3
Upper jaw length	35.7	37.1–40.0	24.0	31.3
Lower jaw length	26.9	34.5–43.5	—	—
Gill opening length	16.4	25.4–30.1	12.7	8.6
Pectoral-fin length	15.2	45.2–51.6	28.3	23.3
% ***in TL***				
Head length	9.7	10.9–11.8	5.8	7.1
Pre-anal length	36.8	32.1–39.4	31.0	33.6
Pre-dorsal length	13.2	9.6–11.2	3.9	4.6
Depth at gill opening	2.2	2.5–3.3	1.8	2.0
Depth at anus	2.3	2.8–3.7	2.2	2.1
**Vertebrate**				
Pre-dorsal	13	11	—	4
Pre-anal	53	49	—	60
Total	150	154	—	183

— No data available.

There are also notable morphological differences between *Cirrhimuraenataiwanensis* sp. nov. and other Indo-West-Pacific congeners. *Cirrhimuraenacalamus* and *C.oliveri* both have significantly smaller heads, measuring 16.6% pre-anal length (PAL) in *C.calamus* (Günther 1870) and 16.4% of PAL in *C.oliveri* (Seale 1910), compared to 26.5% PAL in *C.taiwanensis* sp. nov.; *C.tapeinoptera*, *C.cheilopogon*, and *C.inhacae* have significantly larger pectoral fins, approximately 40–50% HL (Weber and de Beaufort 1916; Smith 1962; Smith and Heemstra 1986) vs 15.2% in *C.taiwanensis* sp. nov.; *C.taiwanensis* sp. nov. also has a shorter pre-anal length, at 36.8% TL, compared to 41.8% in *C.paucidens* (Catania and Fong 2020; Mohapatra et al. 2021). The combined morphological and molecular differences between the new species and all 12 congeners strongly support that the specimen we collected represents a distinct new species, *Cirrhimuraenataiwanensis* sp. nov.

The habitat of the *Cirrhimuraenataiwanensis* sp. nov. is at the estuary of the Langyang River, where the water is brackish year round and has an abundance plankton. The river estuary serves as an important habitat for the Anguilliformes and other brackish and freshwater fish species (Shih et al. 2008; Dahms et al. 2012; Han et al. 2016). The substrate is sandy, and the brackish-water environment is typical habitat for the *Cirrhimuraena* species (Mohanty et al. 2023). With the description of the new species, the ecological importance of the Langyang River estuary is enhanced; this estuary already serves as the type locality of *Lamnostomataiwanensis* and habitat for other anguillid species in Taiwan (Chiu et al. 2018; Lin and Han 2024). In addition, the these new and recently described species suggests that many more unidentified species may be present in brackish waters, which highlight the importance of these environments for biodiversity.

## Supplementary Material

XML Treatment for Cirrhimuraena taiwanensis

## ﻿Additional information

### Data availability

All data that support the findings of this study are available in the main text, and the holotype is deposited in the collection of Biodiversity Research Museum of the Academia Sinica of Taiwan, under registration code ASIZP0082637.
